# Analysis of essential elements in Pito—a cereal food drink and its brands by the single-comparator method of neutron activation analysis

**DOI:** 10.1002/fsn3.95

**Published:** 2014-03-05

**Authors:** Aaron N Adazabra, Apori Ntiforo, Samuel A Bamford

**Affiliations:** 1 Department of Applied Physics, Faculty of Applied Sciences, University for Development StudiesP. O. Box 24, Navrongo, Ghana; 2 National Nuclear Research Institute, Ghana Atomic Energy CommissionP. O. Box LG 80, Legon, Accra, Ghana; 3 Department of Nuclear Science and Application, Graduate School of Nuclear and Allied Sciences, University of GhanaLegon, Accra, Ghana

**Keywords:** Cereal crops, elemental analysis, *k*_0_ standardization method and Ghana's reactor, Pito

## Abstract

Instrumental neutron activation analysis using the single-comparator method has been used for the multielement determination of essential elements in two main types of Pito brewed locally in Ghana. The precision and accuracy of the analytical method was validated and found to be within 10%. In all, eight different brands of Pito depending on the type of cereal crop used in brewing were analyzed for 13 different elements (Al, As, Ba, Cl, Co, Cu, Fe, K, Mg, Mn, Na, Si, and Zn). It was observed that all brands were particularly enriched in nutrient elements Cl, Mg, and K. The rest were generally found in varying concentrations. As these elements are bioavailable in natural form, perhaps in combination with organic constituents, they are likely to be easily digested and assimilated by the human body. Arsenic, a toxic element, was found in insignificant amounts suggesting that it was within safe limits.

## Introduction

It is known that food and food products provide the much needed carbohydrates, proteins, and lipids, dietary fibers, and essential elements required by humans. These elements, in trace amounts, play an important role in metabolic processes and are essential for the general well-being of humans (Kulkarni et al. 2006). This is because essential trace elements in their appropriate concentrations are involved in various very important physiological and metabolic processes and biomedical functions of the body.

Pito, a cereal food drink, is one of such food product that is widely locally brewed and consumed mostly by the populace in Northern Ghana. This food drink is a traditional alcoholic beverage that is prepared from carbohydrate-rich cereal crops such as millet, guinea corn, or maize. It is brewed in many different styles and different taste. Apart from serving as an inebriating drink, Pito is important in fulfilling social obligations such as marriages, naming and burial ceremonies, parties, and other social gatherings (Sanni and Lonner 1993). In northern Ghana, Pito serves as an energy drink in which the youth consume lots of it all day long for their farming activities.

However, it is well-known that even essential elements can be toxic for living organisms when found in concentrations above certain levels (Mclaughlin et al. 1998). Second, Pito is general indiscriminately brewed and sold in hand-rinsed and reused plastic containers and calabashes without undergoing through the Ghana Standards Authority certification test. Third, to the best of the author's knowledge, there is no Ghanaian legislation that establishes the maximum tolerance limits for inorganic contaminants levels in this cereal food drink. More importantly, Pito brewing is generally seen as an art and hence is commonly targeted for adulteration. For these reasons, there is an increasing urgency to determine the inorganic composition in Pito even at very low levels.

Recent advances in technological development of high-resolution radiation detectors such as High-Purity Germanium detectors (HPGe), pneumatic rabbit transfer systems, automated sample handling, Analog-to Digital Converters (ADCs), digitized Multichannel Analyzers (MCAs), cheaper research reactors, and computerized data processing in Neutron activation analysis (NAA) has opened up this new scope to scientists (Adazabra 2010). This analysis is a versatile, highly selective, sensitive, and multielemental analytical method with good specificity, maintaining an important role in precise determination of elemental concentration. These outstanding features have provided the impetus for the method to be used as a robust analytical method for trace elemental determination even at very low levels (Abugassa et al. 1999). At present, INAA utilizing absolute (*k*_0_) standardization is considered to be the most advanced and optimized version of the method (Salma and Zemplen-Papp 1999).

As a result, because of the potential adverse effects associated with the excessive ingestion of food products chemically contaminated there is the growing need for food quality assurance. In addition, the availability of national mineral contents data and acceptable contamination levels in foods products for improved nutrition through better assessment and planning is lacking in Ghana. There is, therefore, the need for routine checks on the elemental composition of the Pito consumed in the country.

### The single-(*k*_0_) comparator method

Even though, the relative (comparator) method of standardization is quite popularly used in most laboratories, the single (*k*_0_) method of standardization in NAA is considered the most convenient technique for multielemental analysis in routine basis due to some difficulties associated with the former. Some of these difficulties include:

- the lack of appropriate standard reference materials that often leads to in-house standards preparation, which may not have the right matrix or homogeneity,
- the frequent abuse of expensive and sometimes not readily available standard reference materials for each analytical process and
- the cumbersome and laborious routine process of sample preparation and irradiation of standards which may not optimize neutron flux with respect to neutron self-shielding each time analysis are to be made.

The method involved the simultaneous irradiation of a sample and a neutron flux monitor such as gold or zirconium and the use of a composite nuclear constant called *k*_0_-factor. Accurate knowledge of nuclear data such are resonance integrals, neutron cross sections for (n, *γ*) reaction, the detector efficiencies and the specific activities of the nuclides in the sample, and the comparator are needed for quantification (Akaho and Nyarko 2002).

Consider an element *i* in a sample of mass *W*, with a specific count rate *A*_*sp,i*_ and detector efficiency at its peak energy, *ε*_*p*_ (*E*_*i*_) that is coirradiated with gold. If the mass of the element in the sample is *m*_*i*_ then, the measured concentration *ρ*, can be found by using the single-comparator equation:


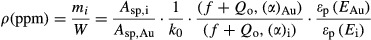
(1)

where *A*_sp,Au_ and *ε*_p_ *(E*_Au_*)* are the specific count rate and detector efficiency for Au, respectively, *k*_0_ is a combination of nuclear constants relative to ^198^Au at its 412 keV *γ*-line, *Q*_o,Au_ (*α*), *Q*_o,i_ (*α*), are the *α*-corrected resonance integral of Au and activated element, i respectively, *f* is the neutron flux ratio and *α* is the epithermal neutron shape factor.

Determination of irradiation site parameters such as *f*, *α* and calculation of *Q*_o_ (*α*) are presented elsewhere (Abugassa et al. 1999; Mumuni 2008; Sogbadji et al. 2010).

The full energy peak efficiency (*ε*) of a high-purity germanium (HPGE) detector may be expressed in the form of a polynomial with respect to the gamma-ray energy (*E*) as (Nix and Scott 1976; Sanderson 1976):


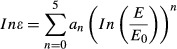
(2)where *ε* is the detection efficiency, *a*_*n*_ represents the fitted parameters, *E* is the energy of the photopeak, and *E*_0_ = 1 keV.

## Materials and Methods

### Sampling

Two main brands of Pito (Frafra and Dagaati) commonly produced were randomly purchased from municipal markets or sales outlets in the Upper East region of Ghana. These were classified into eight categories depending on the type of cereal (early millet, late millet, Guinea corn, or Maize) used for brewing. Table 1 shows the categories considered in this research.

**Table 1:** Pito brands prepared from its constituent cereal food

			Sample categories	
			
No.	Local name	Scientific name	Frafra brand	Dagaati brand
1	Pearl millet	*Pennisetum glaucum*	F1	D1
2	Common millet	*Panicum miliaceum*	F2	D2
3	Guinea corn	*Sorghum* vulgare	F3	D3
4	Maize	*Zea maize*	F4	D4

The quantity of Pito purchased was normally a few liters from which subsamples were taken for analysis. The samples were placed into clean polyethylene containers, freeze and transported to the Neutron Activation Laboratory at Ghana Research Reactor-1 center.

### Samples and standards preparation

Aliquots of each homogenized (by thorough hand-shaken) samples were accurately weighted (in the range of 100–200 mg) into 1.5 mL preclean cylindrical polyethylene vials half-filled with finely grounded sucrose. Four replicates of each of these sample categories were prepared under the same condition for analysis. Also accurately weighed amount of gold solution, prepared from high-purity spectroscopic -grade solution (obtained from SPEX Industries Inc., Canada) was prepared into 1.5 mL precleaned cylindrical polyethylene vials for coirradiation. Again, another four replicate of NISTSRM, Oyster Tissue (1566b) obtained from the National Institute of Standards and Technology, USA, was used to check the accuracy and precision of the analytical method used. All sets of standards and samples were then kept in tubes of 7 mL volume, stacked with cotton and heat sealed for irradiation.

### Irradiation of samples and standards

All vials were sent to the inner (No. 2) irradiation channel of Ghana Research Reactor-1 for irradiation. The neutron flux in this channel is of the order of 5 × 10^11^·ncm^−2^·sec^−1^ on the control console. The neutron flux ratio (*f*) and epithermal neutron shaping factor (*α*) for the same channel had been previously determined (Mumuni 2008; Adazabra, 2010). Detailed specification and design consideration of this reactor have been published in literature (Ahmed et al. 2006; Sogbadji et al. 2010; Alhassan et al. 2010). Irradiation, delay, and counting times were varied depending on the radionuclides present as shown by the irradiation scheme in Table 2.

**Table 2:** Classification of radionuclides based on irradiation time (*t*_i_), half-life (T_1/2_), cooling time (*t*_d_), and counting time (*t*_c_)

Radionuclide	*t*_i_	T_1/2_	*t*_d_	*t*_c_
^28^Al, ^27^Mg, ^139^Ba, ^38^Cl	30 sec to 2 min	2–10 min	1–5 min	300 sec
^56^Mn, ^24^Na, ^42^K, ^31^Si	10 min	Minutes to hours	10 min to 1 h	600 sec
^64^Cu, ^76^As	1 h	Hours to days	1–2 days	1 h
^59^Fe, ^60^Co, ^65^Zn	6 h	Days to years	3 days to 1 month	12–24 h

Thus, radionuclides with longer half-lives (T_1/2_) required longer irradiation, delay, and counting times as shown in Table 2. The converse is true for those with shorter half-lives.

### Counting of samples and standards

A PC-based gamma spectrometry system was used to evaluate the induced gamma activities after the various decay periods stated in Table 2. The system consists of a Canberra N-Type HPGe detector Model GR2518 of relative efficiency of 25% to NaI detector and an energy resolution of 1.8 keV at 1332.5 keV *γ*-ray of ^60^Co, an 8k multichannel analyzer (MCA) Emulation software, for spectral accusation. The other associated electronics consisted of an H.V power supply model 3105, a spectroscopy amplifier model 2020, all manufactured by Canberra Industries Inc. and a NIM power supply unit model PS01-B manufactured by SILENA (Meriden, Australia).

By means of the MCA card, the spectra intensities were accumulated for a preset time. The detection efficiency for the *γ*-ray spectrometer was calibrated with an IAEA mixed standard radionuclide solution containing ^60^Co, ^241^Am, ^109^Cd, ^54^Mn, ^65^Zn, ^85^Sr, ^203^Hg, and ^57^Co. The measured detection efficiencies were fitted by the polynomial function in equation (2) (Osae et al. 1999; Van Do et al. 2009). As a way of reducing uncertainties caused by pile-up effects and random coincidence, the sample-to-detector distance was kept and maintained at 7.2 cm from the top of the detector surface during measurement. Care was taken to account for the counting losses by keeping the dead time around 10% at the start of counting of the samples.

## Results and Discussion

For quick turnaround time and the reduction in personal errors, all the *γ*-spectra obtained were evaluated by a PC version of Hypermet 5. 12 program (Adazabra et al. 2012a,b) from which the activities of the various elements present were ascertained. By using the nuclear data presented by De Corte and Simonits (2003) and the determined detected efficiencies (eq. 2), the ascertained activities were converted to the concentrations of the various elements (eq. 1).

To check the accuracy and precision of the analytical method, four replicates (about 100 mg each) of NIST SRM 1566b (obtained from the National Institute of Standards and Technology, USA), were measured and carefully transferred into 1.5 mL precleaned cylindrical polyethylene vials and irradiated together with the samples and flux monitors under the same experimental conditions.

Table 3 presents the mean measured concentrations of elements in NIST 1566b, Oyster Tissue reference material. The results obtained from this analysis are comparable to the recommended values as shown in the Table 3. The precision and trueness of the counting system was also calculated as a percentage relative standard deviation (%RSD) and the percentage error, respectively, of four replicate measurements of each sample. It is eminently evident that most of these percentages were observed to be less than 10% suggesting high order of accuracy and precision of our data. Therefore, it is presumed that, the measured elemental concentrations in the different brands of Pito should be accurate within ±10% under the same experimental conditions.

**Table 3:** Analysis of NIST SRM oyster tissue (1566b) in mg/kg by NAA

	This work			
		
Element	Mean±SD	RSD (%)	Relative error (%)	Certified values (NIST)
Ba	9.3 ± 0.5	5.38	8.14	8.6 ± 0.3
Ca	795 ± 36	4.53	−5.36	840 ± 20
Cl	5350 ± 110	2.06	4.09	5140 ± 100
Cu	77.8 ± 0.5	0.64	8.66	71.6 ± 1.6
K	6430 ± 120	1.87	−1.38	6520 ± 90
Mg	1115 ± 32	2.87	2.29	1090 ± 23
Mn	17.9 ± 1.8	10.06	−3.24	18.5*
Na	345 ± 46	9.86	4.55	330 ± 53
Rb	3.5 ± 0.1	2.86	7.36	3.26 ± 0.145
S	660 ± 71	10.76	−4.35	690*
Sr	7.6 ± 0.1	1.32	11.76	6.8 ± 0.2
V	0.55 ± 0.02	3.64	−5.17	0.58 ± 0.03
Zn	1380 ± 140	10.14	−3.09	1424 ± 46

RSD, relative standard deviation.

* Recommended or noncertified values.

The measured concentrations of 13 different elements (Al, As, Ba, Cl, Co, Cu, Fe, K, Mg, Mn, Na, Si, and Zn) in eight different brands of Pito are presented in Table 4 as mean values with their standard deviations from quadruplicate analyses. A careful evaluation of the results in Table 4 shows that, irrespective of the type of cereal crop used in brewing or the style, Pito is enriched with a variety of important mineral elements that are essential for the normal development and function of the human body. However, the differences in the concentrations of elements of the same cereal crops species used in the brewing can partially be explained by the different style of brewing. Furthermore, this difference could also result from the different sources of water (which constitute over 95% of the Pito) used for the brewing. More to the point, the philosophy of adding additives to the different brands of the drink preferentially for enhance taste directly accounts for the differences in elemental concentrations of the final food product. That is not all, the determining role played by the environmental conditions (type of soil, pollution, industrial region, use of pesticides, or fertilizers) during the cultivation of the cereal crops may also account for these differences (Serfor-Armah et al. 2003).

**Table 4:** Results of analysis of elements in brands of Pito measured in mg/kg unless otherwise stated

Element	F1	D1	F2	D2	F3	D3	F4	D4
Al	56.81 ± 3.14	37.09 ± 2.70	39.10 ± 3.10	67.86 ± 7.33	25.09 ± 2.19	13.69 ± 1.07	6.18 ± 0.92	36.64 ± 2.94
As	0.16 ± 0.01	BDL	BDL	<0.1	0.211 ± 0.01	0.15 ± 0.01	BDL	BDL
Ba	<0.01	2.78 ± 0.13	3.07 ± 0.161	1.71 ± 0.09	1.35 ± 0.15	<0.01	0.95 ± 0.01	2.14 ± 0.12
Cl (%)	0.47 ± 0.03	0.27 ± 0.02	0.53 ± 0.02	0.49 ± 0.03	0.74 ± 0.04	0.74 ± 0.02	0.57 ± 0.02	0.51 ± 0.02
Co	BDL	<0.01	0.04 ± 0.001	0.06 ± 0.001	BDL	0.03 ± 0.001	0.08 ± 0.001	BDL
Cu	1.08 ± 0.03	1.59 ± 0.04	2.00 ± 0.01	1.14 ± 0.02	1.78 ± 0.03	1.48 ± 0.03	<0.1	1.97 ± 0.04
Fe	71.01 ± 4.13	64.53 ± 3.97	54.82 ± 3.67	67.35 ± 3.51	89.02 ± 4.17	90.07 ± 4.37	78.41 ± 3.75	63.07 ± 2.93
Bk (%)	1.02 ± 0.03	0.21 ± 0.02	0.95 ± 0.02	1.11 ± 0.02	0.61 ± 0.01	0.80 ± 0.03	0.57 ± 0.02	0.51 ± 0.01
Mg (%)	0.11 ± 0.03	0.10 ± 0.003	0.114 ± 0.03	0.12 ± 0.04	0.09 ± 0.001	0.10 ± 0.001	0.07 ± 0.001	0.05 ± 0.001
Mn	9.05 ± 0.25	6.37 ± 0.18	8.94 ± 0.21	7.42 ± 0.19	5.08 ± 0.09	6.14 ± 0.03	4.94 ± 0.26	7.01 ± 0.20
Na	7.95 ± 0.52	8.67 ± 0.64	9.03 ± 0.77	7.98 ± 0.81	14.81 ± 1.73	17.82 ± 1.52	20.01 ± 1.05	22.16 ± 1.07
Si	BDL	0.61 ± 0.01	0.401 ± 0.01	BDL	0.86 ± 0.02	BDL	1.06 ± 0.04	<0.1
Zn	41.45 ± 1.87	40.33 ± 1.96	25.69 ± 1.40	29.51 ± 1.63	12.73 ± 0.97	20.66 ± 0.91	31.72 ± 1.32	27.93 ± 1.57

BDL, below detection limit.

**Figure 1:**
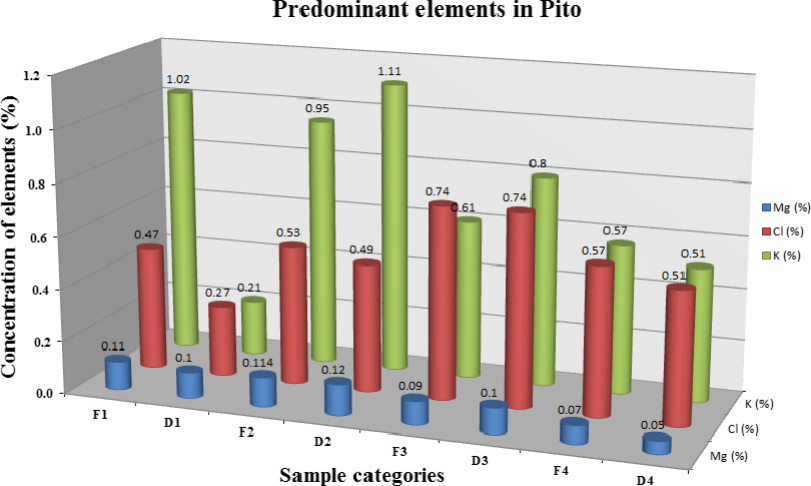
A graph of predominant elements concentrations against brands of Pito.

It is worth noting that, the elements Cl, K, and Mg were found in all brands at the major element concentration levels. Thus, the predominant mineral elements found in all studied brands are generally in an increasing order of Mg < Cl < K as shown in Figure 1.

Mg concentrations ranged from 0.05 ± 0.001% in Pito brand D4 to 0.12 ± 0.04% in Pito brand D2 with those in brands F1, D1, F2, F3, D3, and F4 being 0.11 ± 0.03%, 0.10 ± 0.003%, 0.11 ± 0.03%, 0.09 ± 0.001%, 0.10 ± 0.001%, and 0.07 ± 0.001%, respectively. On the other hand, Cl concentration range from the lowest of 0.27 ± 0.02 (in D1) to the highest of 0.74 ± 0.04 (in F3) with the rest in an increasing order of F1 < D2 < F2 < D4 < F4 < D3. Similarly, the most abundant element K, had concentrations in the range of 0.21–1.11%. Fortunately, these nutrient major elements (Cl, K, and Mg) are present in all brands in bioavailable forms that can easily be digested by our body system (Kumar et al. 2005). These elements are primarily termed electrolytic elements that work synergistically with other ionic elements in maintaining a proper balance of acids, bases, and extracellular fluid of the body (Serfor-Armah et al. 2003; Kumar et al. 2005).

Aside the major elements, other elements of crucial importance present in most of the brands are Al, Ba, Co, Cu, Fe, Mn, Na, Si, and Zn. The relative high concentrations Fe and Al in all brands of Pito to a degree suggest contamination from the milling process and the Aluminum cooking utensils used for brewing of the cereals, respectively. The essentiality of some of these elements for normal biological functions of the body are well-established in literature (Rajurkar and Damame 1997; Naidu et al. 1999; Adazabra et al. 2012a,b). Hence, they are not discussed in this study. Toxicologically, Arsenic (which was not present in all brands) was found in infinitesimal concentrations signifying that it is within safe limits. Other toxic elements such as Pb, Hg, and Cd were not present thereby requiring further analysis in other to establish the overall safety of this rich food drink.

## Conclusion

The elemental composition in Pito is of great importance for its nutritional value as well as estimation of toxic levels. The analysis of elements concentration levels in Pito are very vital for human dietary intake of essential minerals and for the evaluation of human exposure to toxic elements. It is evident that all brands of Pito contain various varying degree of essential elements that are fundamental for the normal physiological and biochemical function of the body. This study could, therefore, be used as a diagnostic tool for the standardization and commercialization of Pito brewing in the Country. Consequently, from the mineralogical point of view, the regular intake of Pito could enhance the health status of the populace.
